# The development and feasibility study of Multidisciplinary Timely Undertaken Advance Care Planning conversations at the outpatient clinic: the MUTUAL intervention

**DOI:** 10.1186/s12904-022-01005-3

**Published:** 2022-07-06

**Authors:** Eline V. T. J. van Lummel, Claudia Savelkoul, Eva L. E. Stemerdink, Dave H. T. Tjan, Johannes J. M. van Delden

**Affiliations:** 1 Department of Intensive Care, Gelderse Vallei Hospital, Willy Brandtlaan 10, 6716 RP Ede, Netherlands; 2 Julius Center for Health Sciences and Primary Care, University Medical Center Utrecht, Utrecht, Netherlands; 3 Department of Anesthesiology, University Medical Center Utrecht, Utrecht, Netherlands

**Keywords:** Advance care planning, Outpatient clinic, Palliative care, Quality of life, End of life, Surprise question, Frailty

## Abstract

**Background:**

Patients still receive non-beneficial treatments when nearing the end of life. Advance care planning (ACP) interventions have shown to positively influence compliance with end of life wishes. Hospital physicians seem to miss opportunities to engage in ACP, whereas patients visiting the outpatient clinic usually have one or more chronic conditions and are at risk for medical emergencies. So far, implemented ACP interventions have had limited impact. Structural implementation of ACP may be beneficial. We hypothesize that having ACP conversations more towards the end of life and involving the treating physician in the ACP conversation may help patient wishes and goals to become more concrete and more often documented, thus facilitating goal-concordant care.

**Aim:**

To facilitate timely shared decision making and increase patient autonomy we aim to develop an ACP intervention at the outpatient clinic for frail patients and determine the feasibility of the intervention.

**Methods:**

The United Kingdom’s Medical Research Council framework was used to structure the development of the ACP intervention. Key elements of the ACP intervention were determined by reviewing existing literature and an iterative process with stakeholders. The feasibility of the developed intervention was evaluated by a feasibility study of 20 ACP conversations at the geriatrics and pulmonology department of a non-academic hospital. Feasibility was assessed by analysing evaluation forms by patients, nurses and physicians and by evaluating with stakeholders. A general inductive approach was used for analysing comments. The developed intervention was described using the template for intervention description and replication (TIDieR).

**Results:**

We developed a multidisciplinary timely undertaken ACP intervention at the outpatient clinic. Key components of the developed intervention consist of 1) timely patient selection 2) preparation of patient and healthcare professional 3) a scripted ACP conversation in a multidisciplinary setting and 4) documentation. 94.7% of the patients, 60.0% of the nurses and 68.8% of the physicians agreed that the benefits of the ACP conversation outweighed the potential burdens.

**Conclusion:**

This study showed that the developed ACP intervention is feasible and considered valuable by patients and healthcare professionals.

**Supplementary Information:**

The online version contains supplementary material available at 10.1186/s12904-022-01005-3.

## Introduction

On average 33–38% of patients nearing their end of life receive non-beneficial treatments in the last six months of their life [1]. According to healthcare professionals (HCPs), advance care planning (ACP) is a promising method to reduce non-beneficial treatments [2]. ACP is defined as "*enabling individuals to define goals and preferences for future medical treatment and care, to discuss these goals and preferences with family and healthcare providers, and to record and review these preferences if appropriate*" [3]. Various ACP interventions have shown to positively influence compliance with end of life wishes [4–6].

Several potential pitfalls for successful ACP interventions may exist. Even if patients have thought about their wishes, most have not shared these with their treating physicians [7], thereby hampering goal-concordant care. This highlights the need for the involvement of HCPs in ACP interventions. A recent review by McMahan et al. studying ACP interventions and their outcomes showed mixed results when analysing quality of care outcomes: the majority of outcomes (congruence and satisfaction with communication and care) were positive, except for goal-concordant care (only 10%) [8]. A potential clarification might be that these ACP interventions focus on documentation, instead of on the process itself, as Brinkman et al. state that ACP interventions focusing on the process may have more effect than documentation of advance directives (ADs) alone [9].

A recent study by Bekker et al. shows that documentation of ACP related items in primary care medical records (as a proxy of ACP implementation) of patients who died non-suddenly was limited, especially in patients with multi-morbidity or organ failure [10]. The importance of documentation is shown in the ACTION trial: a recent randomized controlled trial, investigating an ACP intervention in patients with advanced lung or colorectal cancer in a hospital setting [11]. It entails a scripted ACP conversation, based on the Respecting Choices programme [11]. Korfage et al. did not find an effect on quality of life [12]. However, only 37% of the patients provided the facilitators of the ACP intervention with a copy of their completed My Preference form [12]. When analysing the medical files one year after the conversation, only 10% of the medical files from patients in the intervention group contained ADs [12]. ACP should be a process, however, without wanting to risk premature solidification of the process into a static decision. At the same time, the results of ACTION also stress the importance of documenting the ACP process to be able to achieve goal-concordant care.

There are several reasons underpinning the importance of an ACP intervention at the outpatient clinic. First of all, several studies have shown that hospital physicians focus too much on prolonging life [13–15]. Ahluwalia et al. showed that physicians missed the majority of opportunities (84%) to engage in ACP when analysing whether physicians engaged in ACP during regular outpatient clinic visits when a patient expressed concerns, questions and thoughts regarding their future care [16]. Briedé et al. reported that only 3.1% of the consultations at the internal outpatient clinic involved discussions of future care [17]. This increased to 17.6% after physicians received training on the importance of care decision conversations and training with simulated patients [17]. Second, several barriers for hospital physicians to engage in ACP exist, including lack of communication skills, lack of knowledge concerning ACP, lack of time, cultural differences and fear of medico-legal repercussions [18]. For general practitioners (GPs), one of the potential barriers to engaging in ACP is the lack of knowledge in prognostication for chronically ill patients [19]. This highlights the importance of engagement of ACP by hospital physicians at the outpatient clinic, not solely by GPs. The third reason concerns the nature of patients visiting the outpatient clinic. An outpatient clinic is meant for diagnostic purposes, treatment and/or follow-up for one or more chronic conditions. Hence, patients structurally visiting the outpatient clinic frequently have severe underlying conditions and are therefore at risk of medical emergencies. Hence, if patients visit the outpatient clinic, it is important to timely discuss patient preferences. Fourth, embedding ACP in hospital settings is limited so far [20–22]. The lack of structural implementation of ACP is perceived as a barrier to the improvement of ACP interventions [21]. Hence, structural implementation of ACP may be beneficial.

McMahan et al. suggest that further research is necessary to tailor interventions and outcomes for the local context [8]. To achieve goal-concordant care, ACP interventions may have to be more concrete while still focusing on exploring patient preferences. We hypothesize that having ACP conversations more towards the end of life and involving the treating physician in the ACP intervention may help patient wishes and goals to become more concrete and more often documented. By discussing and documenting patient wishes, end of life care can be organised according to patient preferences.

The benefit of ACP has recently been criticized by Morrison [23] and Morrison et al [24] who stated that ACP fails to improve end of life care. Morrison states that eight steps are needed to achieve the desired outcomes of ACP and argues that scenarios and situations in clinical practices rarely reflect these situations [24]. On the one hand, Morrison acknowledges the importance of documenting and sharing contextual information on patient wishes, on the other hand, he argues that HCPs should not invest in ACP since it does not achieve the desired goals [24]. We argue that reasons for the ineffectiveness of ACP could be overcome by involving HCPs in the process of documentation of contextual information during an ACP conversation, not by not being involved at all as suggested.

The aim of this project was to 1. construct an ACP intervention by following a structured approach, based on insights and evidence, for discussing goals of care in an outpatient clinic setting to facilitate timely shared decision making and foster patient autonomy and 2. determine the feasibility of the intervention.

## Methods

### Setting: ACP in the Netherlands

In the Netherlands, patients are primarily treated by their GP. Hence, the majority of the population receives care from GPs. Patients with one or more severe chronic conditions visit outpatient clinics when specialized care is required. When patients structurally receive care at the outpatient clinic, they may not see their GP regularly. In the Netherlands, awareness of the importance of ACP is increasing. However, ACP is not structurally embedded in ‘usual care.’

### Developing a structured approach

The United Kingdom’s Medical Research Council (MRC) developed a framework to structure the development of complex interventions into a development, feasibility and piloting, evaluation and implementation phase [25]. This structure was used for developing the ACP intervention (Table 1). The development phase consists of three main steps: 1) identifying existing evidence 2) identifying or developing theory and 3) modelling process and outcomes [25]. We followed the recent update of the MRC guideline by Skivington et al. which stresses the importance of understanding the interaction between the intervention and the context in which it is implemented [26].

**Table 1:** Overview of the steps taken in the development and feasibility phase according to the MRC framework [25, 26]

Step 1 – Development *Either developing a new intervention, or adapting an existing intervention for a new context, based on research evidence and theory of the problem* [26]
*1a. Identifying existing evidence* - Identifying existing evidence on the four different phases (preparation, initiation, exploration and action phase) of ACP interventions described by Fahner et al. [27] using meta- and systematic reviews - Identifying key factors, barriers and facilitators for successful implementation of ACP interventions
*1b. Identifying and developing theory and 1c. Modelling process and outcomes* - Discussing barriers and facilitators for successful ACP interventions (results from phase 1a) among stakeholders to 1) identify other (context related) barriers and facilitators, 2) discuss potential impact of the barriers and facilitators and 3) developing theory for previous ACP interventions not being successful - Translation of the input from earlier phases into the different components of the ACP intervention - Development of the intervention materials with stakeholders - Conceptualizing the feasibility study including consensus on the main feasibility criterion (“does the benefit of the ACP conversation outweigh the burden”) (see also Table 2)
Step 2 – Feasibility *Assessing feasibility and acceptability of intervention and evaluation design in order to make decisions about progression to next stage of evaluation* [26]
- Assessing feasibility and acceptability of the MUTUAL intervention by performing a feasibility study consisting of 20 ACP conversations at two outpatient clinics (geriatrics and pulmonology department) - Evaluating the feasibility study using evaluation forms for patients, nurses and physicians - Evaluating the process and outcome of the feasibility assessment in several stakeholder meetings - Finetuning of the MUTUAL intervention and the materials based on suggestions for improvement made by stakeholders

Fahner et al. divide the essential elements of ACP interventions into four different phases [27]. These four phases are used to structure existing evidence for key elements of the ACP intervention. The preparation phase consists of the identification of eligible patients and practical arrangements. The actual conversation is started in the initiation phase, whereas the core part of the conversation consists of an exploration of patient views: the exploration phase. The action phase of ACP interventions can include documenting a summary of the conversation. The existing evidence was identified using meta- and systematic reviews. A feasibility study was performed on the intervention we constructed (and will describe below) to check the feasibility of the developed ACP intervention.

### Description of the intervention

The study population consisted of patients attending the geriatric or pulmonology outpatient clinic of the Gelderse Vallei Hospital in Ede (a non-academic hospital with 300 beds in the Netherlands) between March 2018 and April 2018. All patients attending the outpatient clinic were screened by their treating physician using the surprise question (SQ): “*Would I be surprised if this patient were to die in the next 12 months*?”. Patients were eligible if the treating physician answered “*no”* to the SQ. Subsequently, treating physicians were encouraged to inform and invite patients for an ACP conversation. If a patient agreed to participate, an information folder and preparatory questionnaire were provided, and an ACP conversation was scheduled at the outpatient clinic. A trained nurse practitioner or specialised nurse and the treating physician facilitated the ACP conversation. Conversations were audio recorded. Patients’ family members or other significant persons, hereafter referred to as ‘proxy/proxies’ were encouraged to participate in the conversation. Patients, nurses and physicians were asked to complete an evaluation form directly after the conversation to assess feasibility.

### Feasibility assessment and data collection

The feasibility study aimed to include 10 patients in the geriatrics department and 10 in the pulmonology department. This pragmatic sample size was determined in discussion with the stakeholders and was comparable to another feasibility study (enrolling 30 patients) [28]. We determined feasibility by assessing the acceptability of the intervention and the acceptability of the evaluation of the intervention based on the recommendations for conducting feasibility studies [29–31] and evaluating complex interventions [32]. This study aims to answer the following questions (see Table 2): 1. Are treating physicians able to select frail patients by using the SQ? 2. Are treating physicians willing to inform and invite patients to the ACP intervention? 3. Are patients willing to participate in the ACP intervention? 4. How is the preparation of the ACP intervention evaluated by patients? 5. Is the construction of the ACP intervention feasible? 6. Is documentation of the ACP intervention feasible? and 7. Is the evaluation method of the ACP intervention feasible?

**Table 2:** Feasibility assessment

Element of the ACP intervention	Question
Selection	1. Are treating physicians able to select frail patients by using the SQ? 2. Are treating physicians willing to inform and invite patients to the ACP intervention? 3. Are patients willing to participate in the ACP intervention?
Preparation	4. How is the preparation of the ACP intervention evaluated by patients?
ACP conversation	5. Is the construction of the ACP intervention feasible?
Documentation	6. Is documentation of the ACP intervention feasible?
Evaluation	7. Is the evaluation method of the ACP intervention feasible?

*SQ* Surprise question, *ACP* Advance Care Planning

To answer these questions, the following data were registered for each patient visiting the outpatient clinic until the maximum was reached: responses of physicians to the SQ, whether the patient was informed and invited to the ACP intervention, and whether the patient wanted to participate. Furthermore, participating patients, nurses and physicians received an evaluation form after the ACP conversation to evaluate their experience with the intervention and whether the benefit of the conversation outweighed the burden of the conversation (evaluation forms for patients, nurses and physicians are available in Additional file 1: Appendices B, C and D).

We assessed whether the intervention was feasible in various ways: 1. by analysing the evaluation forms by patients, nurses and physicians and 2. by evaluating with stakeholders and 3. whether the majority of the patients, nurses and physicians agreed that the burden of the ACP conversation outweighed the benefit. The evaluation forms contained both multiple-choice questions (with the possibility for comments) and open-ended questions.

### Analysis

Descriptive statistics were used to summarize patient characteristics. A general inductive approach was used for analysing comments and open-ended questions from the evaluation forms [33]. The description of the final ACP intervention was done based on the TIDieR method and comparable to other research [34, 35].

The study was assessed by the institution’s ethical review board at Gelderse Vallei hospital which judged that this study was outside the scope of the Dutch law on research involving humans. Patients participating in the study provided written informed consent. To minimize the potential harm, patients were offered follow-up visits and support from the palliative care team when deemed of additional value by the facilitating nurse and treating physician. Moreover, patient proxies were encouraged to participate in the ACP conversation for support. Patients were also encouraged to discuss their wishes with their GP, with the GP functioning as an additional supporting network.

## Results

### Identifying existing evidence

To identify the essential elements of the intervention, we first focused on the existing evidence based on the four phases described by Fahner et al [27].

#### Preparation phase

Improving end of life care in patients with chronic diseases is challenging due to their unpredictable course, hence early identification and timely ACP are essential. Timely identification of patients is a commonly mentioned barrier by HCPs, highlighting its importance [36]. Several potentially useful screening methods exist, including the Supportive and Palliative Care Indicators Tool (SPICT), the Radboud Indicators for Palliative Care Needs (RADPAC), and the Palliative Necessities CCOMS-IC (NECPAL), the Gold Standards Framework Indicator Guidance (GSF-PIG) and the SQ [37]. The SQ is an easy to use and intuitive tool to identify patients nearing the end of life and requires HCPs to answer the question: “*Would I be surprised if this patient were to die in the next 12 months?*”. A recent systematic review and meta-analysis of the accuracy of the SQ in predicting death resulted in an estimated sensitivity of 71.4% and an estimated specificity of 74.0% [38]. The SQ has been used successfully as a selection method in other ACP interventions [39].

A commonly mentioned barrier for HCPs to conduct ACP is lack of time [18, 40, 41]. In daily practice, lack of time might be less problematic for nurses compared to physicians. Furthermore, nurses are suggested to play an important role in improving end of life care and implementation of ACP considering 1) patients appreciate discussing life-changing matters with nurses [42] and 2) their intimate role in patient care [43]. Nurse-facilitated ACP interventions have been successfully implemented previously [42, 44].

A good patient-HCP relationship encourages initiating ACP, hereby potentially facilitating an ACP intervention [45]. A systematic review by Risk et al. states that lack of training is a frequently mentioned barrier in initiating ACP for HCPs, whereas providing training enables ACP [46]. A systematic review on the effect of training in ACP shows that training has positive effects on knowledge, attitudes and skills and recommends that training programs for ACP should include training in communication skills [47].

Other barriers for HCPs to initiating ACP conversations include lack of preparedness among patients and caregivers [48] and patient readiness (‘not being ready’) [49]. Sudore et al. studied the level of AD documentation and ACP engagement and conclude that using PREPARE [50] (an interactive website addressing questions on what a patient values most in life and selecting a surrogate decision maker[51]) improved patient preparation, ACP engagement and documentation [50]. To unravel patient readiness in ACP, the content of several ACP conversations was analysed as part of the ACTION trial. Zwakman et al. conclude that patients do not have to be completely ready to discuss all ACP related topics to be able to participate in ACP conversations [49].

To conclude, by using the SQ to initiate ACP, patients potentially approaching the last phase of life will be identified. Having ACP conversations with patients more towards the last phase of life potentially makes the ACP conversation more concrete. Furthermore, promoting factors for a successful ACP intervention include training of facilitators and a good patient-HCP relationship. Patient preparation and patient readiness may also be helpful.

#### Initiation phase and exploration phase

Fahner et al. show that exploring patient views is the main part of an ACP conversation, including an understanding of the disease, perspective on living with the disease and quality of life [27]. Other themes that are regularly addressed include perspectives on death, end of life care, fears, worries and hopes. Exploring patient perspectives on these themes enables patients to formulate goals of care [27]. Pollard et al. state that involving patients in treatment decisions will lead to goal- concordant care and increase patient satisfaction with care and treatment decisions [52].

ACP conversations could also have a relational effect [53] and are suggested to stimulate conversations with proxies [12]. A meta-review by Jimenez et al. highlights the importance of preparing family members to make informed care decisions as potential decision-makers [48]. Weathers et al. conclude that ACP may help surrogate decision-makers in representing patients’ goals of care since ACP enables understanding patient preferences [54]. Moreover, bereaved relatives of patients engaged in ACP feel less anxiety and guilt after their death [4]. Additionally, interventions targeting multiple stakeholders (e.g., patients, caregivers and HCPs) may be more effective in removing barriers to effective end of life communication [55].

The aforementioned evidence shows that exploring patient views on values, illness and goals of care is an important part of ACP. Engagement of multiple stakeholders in ACP and discussing the selection of a surrogate decision-maker may improve goal-concordant care. Furthermore, the presence of patient proxies may improve decision making by the patient representatives. ACP potentially facilitates discussing patient preferences outside the scope of the actual ACP conversation.

#### Action phase

In the ACTION trial, the ACP conversation was conducted by a nurse facilitator and patients were offered the option to complete a ‘My Preference form’. Only 37% of the patients in the intervention group provided their facilitators with their completed form. When analysing the medical files one year after the conversation, only 10% of the medical files from patients in the intervention group contained ADs [12]. A systematic review and meta-analysis by Houben et al. showed a higher completion rate of ADs in patients with ACP compared to the control group [56]. Additionally, patients who completed an AD received more goal-concordant care [56]. Although completing ADs is not the goal of ACP, goals of care must be known and documented to be able to give goal-concordant care.

#### Key factors, barriers and facilitators for successful implementation of ACP interventions

The description by Vleminck et al. of the development of a complex ACP intervention by GPs identified four features that are important for the successful implementation of an ACP intervention including 1) a trained or experienced facilitator 2) a selection process to identify patients eligible for ACP 3) structured and patient-centred ACP discussions and 4) the opportunity to complete ACP documentation [57]. This is supported by Lund et al. who identified that key factors for the implementation of ACP are specially trained staff and the use of a structured approach [40]. Furthermore they state that organizational support is the key success factor in implementing an ACP facilitator training program for HCP in curative care hospital settings [40]. Hence, these are important elements to consider when developing and implementing an ACP intervention successfully.

### Identifying and developing theory and modelling process and outcomes

We modelled our ACP intervention in an iterative process with our stakeholders using the evidence outlined above to formulate theoretical notions and transform these into a preliminary version of the intervention. An important part of the development process consisted of discussing the preliminary format with the research team and important stakeholders including physicians (2 geriatricians and 2 pulmonologists), specialised nurses (2 from the geriatrics department and 3 from the pulmonology department), 2 members of the palliative care team, a communication adviser, an educational expert, administrative supporters and management. To overcome the main barriers to the implementation of interventions we frequently discussed the intervention during the development and feasibility assessment. The different components of the intervention are described below, and the main elements can be found in Table 3.

**Table 3:** Elements of the MUTUAL intervention

1. Timely patient selection	Patients are selected at the outpatient clinic by the treating physician using the surprise question (SQ): “Would I be surprised if this patient were to die in the next 12 months?”. If the physician’s answer to the SQ is “no” the patient is considered eligible for an ACP conversation
2. Preparation of patient and HCP	The treating physician informs the patient about ACP and invites the patient to an ACP conversation. The patient receives an information folder and preparatory questionnaire to encourage the patient to explore his/her ideas on quality of life and preferences of care and to discuss this with proxies. HCPs receive training as preparation
3. Scripted ACP conversation in a multidisciplinary setting	A trained nurse explores patient preferences and goals of care during a scheduled appointment at the outpatient clinic. The first part of the conversation takes approximately 45 min. Subsequently, the physician attends the conversation, and a summary is provided by the nurse
4. Documentation	The nurse composes a letter in which the content of the conversation, including patient preferences for care, is documented. The letter is sent to the patient and his/her general practitioner thus allowing for the process to be continued. By signing the document, it functions as an AD. The treating physician documents the ADs in the electronic healthcare system with a reference to the summarizing letter

For selecting patients for the intervention, we decided to use the SQ as a screening tool to select patients nearing the last phase of life. A patient was considered eligible if the treating physician answered “*no, I would not be surprised…”* to the SQ and if the patient was considered mentally competent. A preparatory questionnaire was developed in cooperation with two experts on quality of life and end of life care from the Netherlands Patients Federation, an association representing more than 200 patient organizations in the Netherlands [58].

This questionnaire was meant to inform, prepare and enable the patient before the actual ACP conversation by helping patients to start the conversation with their proxies and to prepare for the conversation by formulating their thoughts and feelings. It includes questions that enable the HCP to gain insight into the patient’s understanding of his or her illness, experience of health, quality of life and goals of care. Since patient readiness was not a requirement for initiating ACP, HCPs were discouraged to use (their perspective of) readiness of the patient as a criterion not to ask a patient to participate in an ACP discussion.

Preparation for the HCPs consisted of two training sessions, each lasting three hours. The main themes addressed during the training were 1) definition and importance of ACP 2) background information on intensive care treatment and consequences 3) basic training in conversational techniques. The training was tailored to the needs of the participants by exploring the needs in advance.

A conversation manual, containing advice and guidelines to structure the conversation was developed by CS and a communication expert and was based on the steps of Manu Keirse [59]. The script aims to support the facilitator in exploring patient perspectives during the conversation. The conversation manual consists of seven steps including 1) introduction 2) quality of life 3) goals of care 4) scenario sketching 5) choosing a representative 6) conclusion and 7) documentation. A ‘lifeline’ [60] (see Additional file 1: Appendix A), (a straight line drawn on a piece of paper, with the start representing the beginning of life and the end representing the end of life) was presented as a tool for starting and concretizing the conversation.

A trained nurse and the treating physician facilitated the ACP conversation. Patient proxies were able to participate. The facilitating nurse started the conversation by exploring the patient’s wishes and preferences. During the exploration phase patient values, quality of life, fears and worries and goals of care were explored. Furthermore, plausible future scenarios tailored to the patient’s illness were discussed to see whether the patient was able to oversee the consequences of certain decisions. Eventually, preferences for future medical care and treatment limitations were discussed.

After approximately 45 min the physician joined the conversation. The nurse gave a summary of the conversation. This allowed the patient to check if his/her wishes were understood correctly. The treating physician could answer questions and verify preferences for future medical care and treatment limitations if applicable.

The content of the conversation was documented by the nurse in a summarizing letter. Treatment preferences were documented in the summary of the conversation and the electronic healthcare system (including a reference to the summarizing letter). The summarizing letter was sent to the patient for verification and his/her GP. By signing the document, the document functioned as an AD. Furthermore, the patient was stimulated to discuss his/her preferences with his/her GP. Thus, the process of ACP could be continued if so desired.

### Feasibility study

A feasibility study was performed to test the feasibility of the developed intervention. In total 20 patients (baseline characteristics are presented in Table 4), 5 specialized nurses (2 from the geriatrics department and 3 from the pulmonology department), 1 geriatrician and 3 pulmonologists participated in the feasibility study. Results are presented in Table 5.

**Table 4:** Baseline characteristics of patients participating in the feasibility study

	**Geriatrics (*n* = 10)**	**Pulmonology (*n* = 10)**
Mean age, in years (range)	78.8 (65–95)	65.8 (49–76)
Female sex (%)	7 (70%)	6 (60%)
Primary diagnosis (n)	Parkinson’s disease (*n* = 3) Dementia (*n* = 3) Mild Cognitive impairment (*n* = 3) Severe osteoporosis (*n* = 1)	COPD Gold IV (*n* = 5) Lung cancer (*n* = 5)

**Table 5:** Results feasibility assessment

	**Pulmonology department**	**Geriatrics department**
**Acceptability of the intervention**		
1. Are treating physicians able to select frail patients by using the SQ?	759 visits from 755 individual patients were screened in 55 days. In 52 of the 755 (6.9%) patients the pulmonologist answered the SQ with “no.” One patient was included after a resident answered the SQ with “no”	65 visits from 54 individual patients were screened in 20 days. In 34 of the 54 (63.0%) patients the geriatrician answered the SQ with “no.” One patient was included despite the answer to the SQ being “yes.”
	All physicians experienced answering the SQ as positive	
2. Are treating physicians willing to inform and invite patients to the ACP intervention?	36/52 (69.2%) of the patients were not informed	10/34 (29.4%) of the patients were not informed
3. Are patients willing to participate in the ACP intervention?	11/17 (64.7%) of the invited patients wanted to participate	13/25 (52.0%) of the invited patients wanted to participate
4. How is the preparation of the ACP intervention evaluated by patients?	The information folder is perceived as positive by 11/18 (61.1%) of the patients and neutral by 4/18 (22.2%) of the patients. Three patients answered negatively, one did not receive the preparation, one explained it was not very useful and one stated that it contained too much information The preparatory questionnaire is perceived as positive by 14/19 (73.7%) of the patients and neutral by 3/19 (15.8%) of the patients. Two patients answered negatively, one did not receive the preparation and one explained that the questions were hard to answer	
5. Is the construction of the ACP intervention feasible?	The physician was able to join the ACP conversation in 18/20 (90.0%) of the conversations. 18/19 (94.7%) of the patients agreed that the benefits of the ACP conversation outweighed the potential burdens compared to 12/20 (60.0%) of the nurses and 11/16 (68.8%) of the physicians	
6. Is documentation of the ACP conversation feasible?	In all cases, a reference was made to the more extensive letter in which the conversation was documented. 13/16 (81.3%) of the nurses experienced documentation as positive and 3/16 (18.8%) as neutral. 10/11 (90.9%) of physicians experienced documentation as positive, one as neutral. There were no negative responses	
**Acceptability of the evaluation of the intervention**		
7. Is the evaluation method of the ACP intervention feasible?	The response rate was 19/20 (95.0%) for patients, 20/20 (100%) for nurses and 16/18 (88.9%) for physicians	

*SQ* Surprise question, *ACP* Advance Care Planning

In the geriatrics department, 65 visits from 54 individual patients were screened in 20 days including 10 patients. The geriatrician answered the SQ with “*no*” in 34 of 54 patients. One patient was included despite the answer to the SQ being “yes”. In 10 cases the geriatrician did not inform the patient. Of the informed patients, 12 patients did not want to participate and 3 were not able to participate. In the pulmonology department, 759 visits from 755 individual patients were screened in 55 days including 10 patients. The pulmonologist answered the SQ with “*no*” in 52 of 755 patients. One patient was included after a resident answered the SQ with “no”. 36 patients were not informed of the ACP intervention. Of the informed patients, 6 patients were not willing to participate and one patient was not able to participate.

In 2/20 conversations the physician could not join the conversation due to organisation difficulties (in one conversation a resident joined instead). In total 18/20 conversations were audio-recorded. On average the ACP conversation lasted 59 min for the geriatrics department (range 35–83) and 53 min for the pulmonology department (range 39–66).

Evaluation forms were completed by patients in 19/20 (95.0%) of the conversations, by nurses in 20/20 (100%) of the conversations and by treating physicians in 16/18 (88.9%) of the conversations. Overall, patients, nurses and physicians considered the ACP intervention valuable. This was reflected in the comments made on the evaluation forms (Table 6). 18/19 (94.7%) of the patients, 12/20 (60.0%) of the nurses and 11/16 (68.8%) of the physicians agreed that the benefits of the ACP conversation outweighed the potential burdens. Since the majority of the participating patients, nurses and physicians agreed that the benefits outweighed the burden, the intervention was assessed as feasible. One patient was not sure what to answer, while at the same time evaluating the conversation as very positive. In 8/20 (40.0%) of the conversations, the facilitating nurses did not agree the benefits outweighed the burden of the conversation, mainly answering they were not sure (e.g., they mentioned that benefits were not clear or stated that time investment was high). Physicians remarked that time investment was high and that it was more difficult to judge whether the benefits outweighed the burden of the conversation if patients already had conversations discussing treatment limitations.

**Table 6:** Illustrative comments by patients, nurses and physicians

Topic	Quotes
Information folder	**Patient**: “clear”, “good way of preparing”, “not very useful, too extensive”, “too much information”, “confronting”, “confronting, had not thought about this”, “a lot of important issues are addressed”
Preparatory questionnaire	**Patient:** “very positive”, “difficult to answer”, “good points to consider”, “confronting but also supporting”, “intense”, “in a nice way several issues were addressed”, “eventually positive”
Conversation manual	**Nurse**: “great to have questions as guideline”
Interaction nurse / physician (multidisciplinary setting)	**Nurse**: “physician concretised to medical decisions”, “physician outlined great examples”, “physician clarified things” **Physician**: “complementary”, “clear summary, nurse gave patient opportunity to add things if necessary”
How did you experience this conversation?	**Patient**: “good”, “important and sad”, “very positive”, “useful”, “clarifying”, “good and open”, “emotional”, “confronting”, “a revelation”, “I did not experience problems” **Nurse**: “very good”, “open conversation about life and death and what matters most to the patient”, “important issues were discussed”, “difficult since patient did not know what the conversations was about and expectations were not clear”, “hard work” **Physician:** “helped clarifying patient wishes”, “good to discuss these topics with the patient”, “difficult since it was hard to clarify patient wishes”
Did this conversation help you to express your wishes?	**Patient**: “more clarity”, “yes”, “yes I have been reassured”, “certainly”, “I still need time to think about it”
What did the conversation yield?	**Nurse**: “well informed decision on treatment”, “a lot, patient was relieved” “clarity concerning patient wishes”, “clarity concerning euthanasia”, “peacefulness”, “patient realised that ageing and end of life is getting closer and that discussing this is important”, “insight in patient fears”, “clarity concerning resuscitations and ICU treatment” **Physician:** “concrete decisions”, “change of medical decisions, supported by all stakeholders”, “awareness”, “the intervention stimulated the patient to think about her wishes and concrete treatment decisions”, “gratefulness and reassurance with patient”, “clear agreements”, “fears concerning suffocating taken away”, “good preparation for potential medical emergencies”
Did the benefits outweigh the burden?	**Patient**: “definitely, very important to discuss treatment in the last stage [of life]”, “yes, 100% worth it”, “there was no burden”, “certainly”, “I don’t know” **Nurse**: “there was no burden”, “definitely”, “not sure, time investment is high”, “not sure, no clear results” **Physician**: “certainly”, “I think the conversations are very important, however time investment is high”, “not sure”

The information folder and questions from the preparatory questionnaire were considered “*confronting”* and *“difficult to answer”* by patients, nevertheless considered of additional value, referred to as “*good way of preparing*” and revealing “*good points to consider*”. Overall, patients were positive about the ACP conversation, describing the ACP conversations as “*useful”*, “*clarifying”*, and “a revelation*”.* Even if patients evaluated parts of the ACP intervention (information folder, preparatory questionnaire and/or the conversation itself) as hard, confronting or emotional, they almost all stated that the benefits of the conversation outweighed the potential burden. No negative comments were made regarding the ACP conversation.

Nurses described the ACP conversations as “*an opportunity for patients to tell their story*” and “*intense but useful”.* Furthermore, they highlighted the importance of knowing what intensive care treatment entails to be able to accurately inform the patient and their proxies. Using the lifeline proved insightful in understanding patient perception of his/her illness. The summary given (when the physician joined the conversation) was considered to be useful for confirming whether patient preferences were interpreted correctly. Documentation of the ACP conversation was experienced as clear, but also as time-consuming, especially at the start of the intervention.

The physicians evaluated that the SQ was “*easy to use”* and “*accommodating”* for selecting patients. The multidisciplinary setting was experienced as valuable by facilitating nurses and physicians. 12/20 documented treatment preferences were either new or different after the ACP intervention compared to earlier documented preferences. In all cases, a reference was made to the more extensive letter in which the conversation was documented. The length of these letters was one page on average.

### Description of the MUTUAL intervention

Based on the feasibility study the intervention was fine-tuned. Minor changes were made, based on the evaluation forms from patients, nurses and physicians and discussions with stakeholders. Textual changes were made in the information folder and preparatory questionnaire. The conversational manual was adapted based on experiences from the facilitating nurses. Adaptions consisted of rearrangement of the structure and adding illustrative sentences. Additionally, the SQ was integrated into the electronic healthcare system to facilitate screening. The summarizing letter was also incorporated into the electronic healthcare system to facilitate sending the summarizing letter to the patient and GP. The feasibility study highlighted the importance of training on the job. Hence, the final intervention includes an experienced facilitator joining the first two ACP conversations of every starting facilitator. Key components of the MUTUAL intervention (MUltidisciplinary Timely Undertaken Advance Care PLanning) consist of 1) timely patient selection 2) preparation of patient and HCP 3) a scripted ACP conversation in a multidisciplinary setting and 4) documentation. The description of the intervention according to TIDieR can be found in Table 7 [34].

**Table 7:** Description of the MUTUAL intervention according to TIDieR [34]

	**Timing**	**Intervention component**	**What** *Intervention activities, procedures and processes*	**How** *Mode of delivery*	**Who** *The intervention provider(s) and participants*	**Materials** *Resources/tools that support the intervention activities*
**1) Preparing for start of intervention**						
**Engagement, selection and preparation of stakeholders**	*6 months prior to start*	***Selection research team***^a^	*The research team and trainers should be selected*	NA	*The research team should include persons with experience within ACP, palliative care, conversational techniques, intensive care and implementation of interventions*	NA
	6 months prior to start	**Initiating ACP intervention**	The research team should engage important stakeholders including nurses, physicians, supporting staff and management by discussing experiences within healthcare and consequences of absent goals of care when having to make a decision. Informing potential stakeholders of the goals and logistics of the intervention. Determining necessity and support for implementation of the intervention	In group meetings / staff meetings	1) Research team 2) Stakeholders	NA
	5 months prior to start	**Selection of participating specialties and HCPs**	Informing HCPs of the goal of the intervention, inviting HCPs to think about implementation of the intervention within their own specialty	Both individually and group meetings	1) Research team 2) HCPs from different specialities	Necessary competencies were listed by the research team with help of an educational expert and specialised nurse from the palliative care team
**2) Components of the MUTUAL intervention**						
**Preparation**	After all preparations for the intervention are finalised	**1. Patient selection**	Selecting, informing and inviting patients for an ACP conversation	Selection of patient using the surprise question at the outpatient clinic	1) Treating physician	Surprise question: “*Would I be surprised if this patient were to die in the next 12 months*?”
	Training part 1 – before start of the intervention Training part 2 – during the first two conversations	**2a. HCP preparation**	The first training part consists of two sessions of three hours consisting of different elements including 1) Definition, goals and importance of ACP 2)Background information on intensive care treatment and consequences 3) Training in conversational techniques 4) Logistics of the ACP intervention The second part of the training consists of training on the job. An experienced facilitator joins the first two ACP conversations of the facilitating nurse, which are (de)briefed in a structured manner	Part 1 – in a group Part 2 – individually	Part 1 1) Research team provides training 2) Participants: nurses, treating physicians and supporting staff Part 2 1) Experienced facilitator 2) Facilitating nurse	Training material includes 1) Presentation on definition, goals and importance of ACP 2) Presentation on Intensive Care 3) The conversational manual and presentation on conversational techniques
	After the patient has been informed and invited	**2b. Patient preparation**	The treating physician informs the patient about ACP and invites patient to an ACP conversation during an appointment at the outpatient clinic. The preparatory questionnaire and information folder are handed out by supporting staff	NA	1) Treating physician 2) Patient and proxy	1) Information folder explaining goals of the conversation 2) Preparatory questionnaire for patient and proxies to prepare for the conversation. The questionnaire includes questions on the themes of the conversation concerning: understanding of illness, experience of health, quality of life, future and goals of care
**Initiation and exploration**	Scheduled 45’ appointment at outpatient clinic	**3a. ACP conversation – part 1**	Conversation exploring patient preferences and goals of care during a scheduled appointment at the outpatient clinic. First part takes approximately 45 min	Personalised	1) Trained nurse facilitator 2) Patient and proxy	Conversation manual consisting of seven steps: 1) Introduction of topic 2) Quality of life 3) Goals of care 4) Scenarios 5) Representative
	Scheduled 15’ appointment at outpatient clinic	**3b. ACP conversation – part 2**	Consecutive conversation exploring patient preferences and goals of care. Second part takes approximately 15 min and starts with a summary provided by the facilitating nurse	Multidisciplinary	Multidisciplinary setting 1) Trained nurse facilitator 2) Treating physician 3) Patient and proxy/proxies	Conversational manual step: 6) Summary and conclusion
**Action**	Directly after conversation	**4a. Documentation for patient and general practitioner**	The nurse documents the content of the conversation using the format within electronic healthcare system	Individually	1) Nurse documents patient preferences; 2) The patient receives letter 3) GP receives letter	Conversational manual step: 7) Documentation Standardised letter consisting of several components: A) Quality of life and future expectations B) Preferences and goals of care C) Social support D) Conclusion E) Representative F) Treatment registration
	Directly after conversation	**4b. Documentation in health care system**	The treating physician documents ADs using the registration for treatment limitations in the healthcare system	Individually	1) Treating physician	ADs in health care register

*ACP* Advance Care Planning, *MUTUAL* Multidisciplinary Timely Undertaken Advance Care Planning, *TIDieR* Template for intervention, description and replication, *ADs* Advance directives, *NA* Not applicable

^a^Research team involved in this study consisted of an ethicist, intensivist, researcher and experienced nurses from the palliative care team

## Discussion

### Main findings

In this paper, we described the development process and feasibility assessment of the MUTUAL intervention: a multidisciplinary timely undertaken ACP intervention at the outpatient clinic. The final intervention, developed with patients and HCPs, consists of 1) timely patient selection 2) preparation of patient and HCP 3) a scripted ACP conversation in a multidisciplinary setting and 4) documentation to allow follow-up. We developed an information folder and preparatory questionnaire together with the Netherlands Patients Federation to prepare patients and proxies for the ACP conversation. Additionally, we developed a structured conversation manual and training for HCPs to guide the exploration of patient preferences. It is commonly known that is a challenge to routinely incorporate ACP in clinical practice. Hence, we considered common barriers and facilitators in ACP implementation in the development of this ACP intervention by reviewing the existing literature and engaging all stakeholders from the beginning. The feasibility assessment showed that 94.7% of the patients, 60.0% of the nurses and 68.8% of the physicians agreed that the benefits of the ACP conversation outweighed the potential burdens. Since the majority of the participating patients, nurses and physicians agreed that the benefits outweighed the burden, the intervention was assessed as feasible. Even if patients evaluated parts of the ACP conversation (information folder, preparatory questionnaire and/or the conversation itself) as hard or confronting, they almost all stated that the benefits of the conversation outweighed the burden. Of the HPCs that did not agree that the benefits outweighed the burden, most were not sure since the benefits were not clear, the time investment was high, or they were not sure of the added value if patients already had conversations discussing treatment limitations. The numbers needed to screen and select 10 patients in the geriatrics department and the pulmonology department were considerably different (54 patients versus 755 patients). Multiple factors could have contributed to these differences, including 1) differences in (predictable) prognosis between patients (e.g., a COPD patient compared to an oncology patient) 2) doctor related barriers to inviting patients and 3) patient-related barriers to engaging in ACP conversations.

### Strengths and limitations

A strength of this study is that multiple stakeholders, including patients, were involved in the development of the intervention to overcome the main barriers to implementation. Furthermore, this intervention has been described in detail using an evidence-based method to improve applicability in other settings [34]. This study has several limitations. First of all, due to the scope of all possible elements of the intervention, the existing evidence was solely based on systematic reviews and meta-reviews. Second, this study was performed in a single centre which may limit generalizability to other institutions. However, the elaborate description of the development process and the eventual intervention allows adaption to the local context. Third, discussions with stakeholders have not been audio recorded or thematically analysed, possibly leading to an emphasis on specific elements due to individual perspectives. Fourth, selection bias could have occurred in patient selection. To conclude, follow-up was not structurally implemented in the developed intervention, whereas ACP is a continuous process and goals of care should be regularly discussed and updated. Possible triggers for ACP could be hospitalisation, a new diagnosis or deterioration of the patient’s condition. We expect that both patients and HCPs will more easily address ACP after the MUTUAL intervention during regular visits to the outpatient clinic.

### Implications for practice

Ideally, ACP is a continuous process involving different moments in time and different professionals in different settings. Successful ACP interventions potentially improve shared decision making by contributing to the shift from a biomedical perspective towards a person-centred perspective that is needed within shared decision making as suggested by Verberne et al. [61]. We believe the MUTUAL intervention contributes to this shift since it aims to overcome barriers for successful implementation of ACP. Given that stakeholders were engaged throughout the development process and the intervention was considered feasible and valuable by patients and HCPs, this is reassuring for the successful implementation of the intervention. We hypothesize that the key elements of our intervention may be used in various settings. Development and implementation studies of ACP interventions have been done in nursing homes, [35] in general practice [57, 62, 63] and the hospital setting [12]. As stated before, ACP has been criticized for its lack of effect and limited implementation. By introducing the treating physician towards the end of the conversation we hypothesized that goals of care would become more concrete and more often documented. A possible limitation of a physician entering the conversation is the medicalization of the conversation, possibly shifting the focus of the conversation to treatment options. More in-depth research is needed to analyse the content of the conversations and the possible effects of the multidisciplinary setting. Further research is also needed to evaluate barriers and facilitators for the structural implementation of the developed ACP intervention.

## Conclusion

We developed an ACP intervention for patients approaching the last phase of life, enabling a more concrete ACP conversation to stimulate more goal-concordant care. Key components of the developed intervention consist of 1) timely patient selection 2) preparation of patient and HCP 3) a scripted ACP conversation in a multidisciplinary setting and 4) documentation. This study showed that the developed ACP intervention is feasible and considered valuable by both patients and HCPs. Further research is needed to evaluate structural implementation.

## Supplementary Information

**Additional file 1: Appendix A.** Lifeline. **Appendix B.** Evaluation form for patients. **Appendix C**. Evaluation form for nurses. **Appendix D.** Evaluation form for physicians.

## Data Availability

The datasets analysed during the current study are not publicly available due to participant privacy reasons but are available from the corresponding author on reasonable request.

## Abbreviations

ACP: Advance Care Planning
AD: Advance Directives
GP: General Practitioner
HCP: Healthcare Professional
MUTUAL: MUltidisciplinary Timely Undertaken Advance Care PLanning
MRC: Medical Research Council
TIDieR: Template for intervention description and replication
SQ: Surprise Question (“*Would I be surprised if this patient were to die in the next 12** months*?”)
