# Supplementary material for: The development and feasibility study of Multidisciplinary Timely Undertaken Advance Care Planning conversations at the outpatient clinic: the MUTUAL intervention
Source: BMC Palliat Care. 2022 Jul 6;21:119. doi: 10.1186/s12904-022-01005-3 (PMC9258045; doi:10.1186/s12904-022-01005-3)
Supplement: Supplementary file 1 — Additional file 1: Appendix A. Lifeline. Appendix B. Evaluation form for patients. Appendix C. Evaluation form for nurses. Appendix D. Evaluation form for physicians. [file 12904_2022_1005_MOESM1_ESM.docx]

# Appendix A – lifeline

**Beginning of life**

**End of life**

**Appendix B – Evaluation form for patients**

| - | +/- | + |
| --- | --- | --- |
| Explanation: | | |

1. How did you experience the introduction of the conversation by the doctor?
2. How did you experience the information folder explaining the conversation?

| - | +/- | + |
| --- | --- | --- |
| Explanation: | | |

1. How did you experience the preparatory questionnaire?

| - | +/- | + |
| --- | --- | --- |
| Explanation: | | |

1. How did you experience the conversation with the nurse?

| - | +/- | + |
| --- | --- | --- |
| Explanation: | | |

1. How did you experience the conversation with the physician?

| - | +/- | + |
| --- | --- | --- |
| Explanation: | | |

1. How did you experience this conversation?
2. Did this conversation help you to express your wishes?
3. Do you think the burden of the conversation outweighs the benefits of the questions asked during the conversation?

**Appendix C – Evaluation form for nurses**

1. How did you experience the preparation for the conversation?

| - | +/- | + |
| --- | --- | --- |
| Explanation: | | |

1. How did you experience the conversation?

| - | +/- | + |
| --- | --- | --- |
| Explanation: | | |

1. How did you experience the interaction with the doctor?

| - | +/- | + |
| --- | --- | --- |
| Explanation: | | |

1. How did you experience documentation of the conversation?

| - | +/- | + |
| --- | --- | --- |
| Explanation: | | |

1. What did the conversation yield?
2. Do you think the burden of the conversation outweighs the benefits of the questions asked during the conversation?

**Appendix D – Evaluation form for physicians**

1. How did you experience answering ‘*the surprise question’*?

| - | +/- | + |
| --- | --- | --- |
| Explanation: | | |

1. How did you experience introducing the conversation?

| - | +/- | + |
| --- | --- | --- |
| Explanation: | | |

1. How did you experience the conversation?

| - | +/- | + |
| --- | --- | --- |
| Explanation: | | |

1. How did you experience the interaction with the nurse in the conversation?

| - | +/- | + |
| --- | --- | --- |
| Explanation: | | |

1. How did you experience documentation of the conversation?

| - | +/- | + |
| --- | --- | --- |
| Explanation: | | |

1. What did the conversation yield?
2. Do you think the burden of the conversation outweighs the benefits of the questions asked during the conversation?
